# Safety and Efficacy of Photodynamic Therapy in the Treatment of Circumscribed Choroidal Hemangioma: A Systematic Review

**DOI:** 10.7759/cureus.50461

**Published:** 2023-12-13

**Authors:** Waleed M Alshehri, Badr O AlAhmadi, Fatima Alhumaid, Mohammed S Khoshhal, Zakaria Y Khawaji, Husain AlHabuobi, Abdulrahman M Alosaimi, Abdulmajeed Alkhathami, Jehad Alorainy

**Affiliations:** 1 Ophthalmology, King Salman Bin Abdulaziz Medical City, Madinah, SAU; 2 Ophthalmology, Prince Mohammed bin Abdulaziz Hospital, Madinah, SAU; 3 Ophthalmology, Imam Abdulrahman Bin Faisal University, Alkhobar, SAU; 4 Ophthalmology, Ohud Hospital, Madinah, SAU; 5 Ophthalmology, Taibah University, Madinah, SAU; 6 Ophthalmology, Majmaah University, Al Majma'ah, SAU; 7 Ophthalmology, University of Bisha, Bisha, SAU; 8 Ophthalmology, King Saud University, Riyadh, SAU

**Keywords:** efficacy, safety, choroid, exudative retinal detachment, photodynamic therapy, circumscribed choroidal hemangioma

## Abstract

Circumscribed choroidal hemangioma (CCH) is a sort of non-malignant hamartomatous tumor that occurs in the choroidal layer of the eye. It is a rare condition that affects people between their second and fourth decades of life, leading to significant deterioration of vision. One of the most catastrophic consequences of CCH is exudative retinal detachment (ERD), which has a severe impact on vision. This review aims to comprehensively assess the safety and efficacy of photodynamic therapy (PDT) using verteporfin as a therapeutic approach.

Using the eligibility criteria, we analyzed the findings of 18 published articles from PubMed, Web of Science, Scopus, and Cochrane. The standard PDT protocol was used in all included studies, except two (one used half-dose, the other one used the double-dose) with an average of 1-2 sessions. PDT induced substantial tumor regression, with a mean thickness range from 0 to 2.3 mm. However, this contrasted with a previous study that reported a thickness of 3.46 mm as an indication of PDT failure. The mean tumor diameter varied from 4.8 mm to total tumor flattening. A suboptimal effect with a mean diameter ranging from 6mm to 8mm was found in two clinical studies. Significant improvement in vision was observed during the last follow-up, ranging from a normalization of Best Corrected Visual Acuity (BCVA) 20/20 to 20/80; counting finger vision persisted in two patients even after treatment. PDT successfully achieved complete subretinal fluid (SRF) resolution in 14 studies and resolved ERD in nine articles. Most studies did not report serious adverse events, but some reported macular atrophy, microcystic degeneration of the retina, transient visual disturbances, Retinal pigmented epithelium (RPE) metaplasia, and cystic degeneration of the retina.

This systemic review demonstrated PDT's effectiveness and safety as a first-line management modality for CCH. Photodynamic therapy efficiently induced tumor regression, resulting in a notable reduction in both tumor diameter and thickness, with optimal efficacy to improve vision and resolution of the consequences of CCH, such as SRF and ERD.

## Introduction and background

Choroidal hemangioma (CH) is a non-malignant vascular tumor that emerges either in a circumscribed or diffuse, which is commonly related to Sturge-Weber syndrome. This condition is characterized by discretely smooth, oval-shaped, orange-red masses usually found posterior to the equator in the macular and peripapillary regions [1].

This non-malignant condition has the potential to cause severe visual impairment. Exudative activity facilitates the progression of macular edema and/or exudative retinal detachment (ERD), eventually leading to the loss of photoreceptors. There is usually no need to treat small asymptomatic tumors. However, it should be regularly monitored for signs of progression or exudation. If indicated, a variety of treatment modalities can be instituted for the management of CH, including cryotherapy, laser photocoagulation, proton beam radiation therapy, external beam radiation therapy, transpupillary thermotherapy (TTT), or plaque brachytherapy [2]. These modalities have proven their efficacy in stabilizing or improving exudative activity but not so much in stabilizing or restoring visual function due to their inability to avoid causing damage to adjacent eye structures, for example, Bruch's membrane and the neurosensory retina. Furthermore, the results of all of these treatments were less favorable when the fovea was involved [3].

Photodynamic therapy (PDT) is considered one of the treatment modalities for maintaining neuroretinal structures selectively through a vasocclusive modality. Highly vascularized choroidal tumors have responded completely to experimental implantation in the suprachoroidal space [4]. It has the advantage of selectively treating pathologic vessels, causing minimal collateral retinal damage, improving visual acuity, and completely subsiding the subretinal fluid (SRF) in circumscribed choroidal hemangioma (CCH). In addition, PDT offers a low complication rate, high visual outcomes, a low recurrence rate, and low treatment-associated morbidity [5-8].

There are a variety of indications for PDT with verteporfin that include the treatment of choroidal neovascularization in myopia, age-related macular degeneration, and presumed ocular histoplasmosis syndrome, as it offers selective laser stimulation of choroidal vascularized structures [9-12]. In CCH, PDT is considered the primary line of management modalities using a variable number of protocols that vary in timing of infusion, dosages, levels of energy, timing of application of laser, spot sizes, single or overlapping spots, and repeating or not repeating the treatment [3,13]. 

This systematic review aims to comprehensively examine the existing body of literature within medical databases investigating PDT's safety and effectiveness utilizing verteporfin as a therapeutic approach for circumscribed Choroidal hemangioma.

## Review

Methods

Study Design

**Figure 1:**
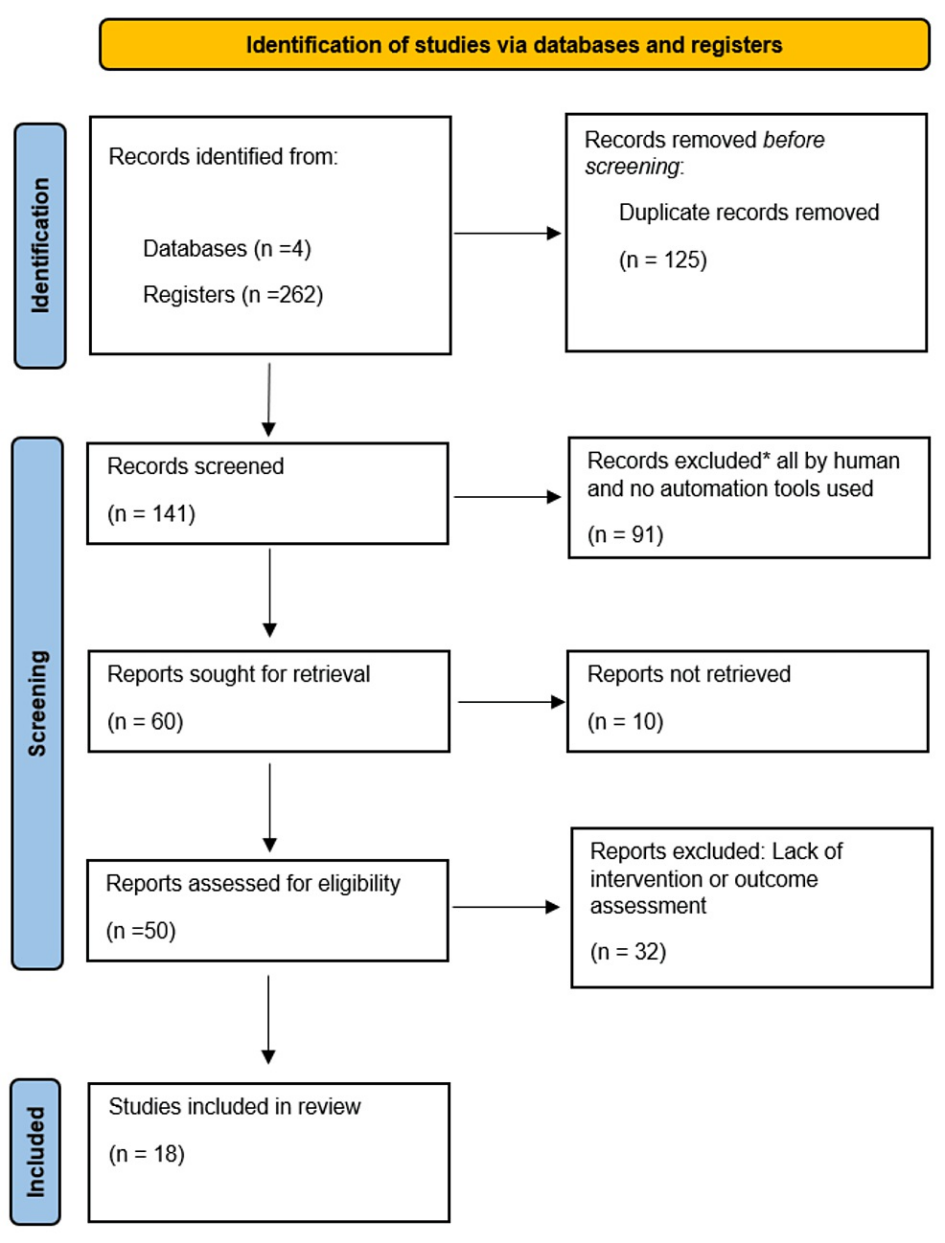
Flowchart depicting the selection of articles

We have established a systematic review to explore the available studies in the literature that have assessed or reported the efficacy of PDT in the treatment of CCH. It was carried out according to the Preferred Reporting Items for Systematic Reviews and Meta-Analyses (PRISMA) guidelines, as shown in Figure 1.

Search Strategy

The search was carried out systematically in June 2023 using four databases: PubMed, Web of Science, Scopus, and Cochrane. Search terms included "circumscribed choroidal hemangioma" OR "CCH "AND "photodynamic therapy" OR "PDT". This resulted in the identification of 262 results. After removing duplicated records, the titles and abstracts of identified results were screened based on inclusion and exclusion criteria by 7 independent reviewers. The full texts of the studies that met the criteria were recovered and screened by the same reviewers. Any conflicts between authors were discussed and solved. This resulted in 18 eligible studies included in our review.

Eligibility Criteria

The inclusion criteria comprised studies written in English, assessed humans only, and examined the efficacy of PDT in treating CCH. All published study designs are included except case reports and literature reviews. On the other hand, we excluded any papers not published in English, inaccessible results, duplicates, and non-human studies, studies that assessed the efficacy of PDT combined with other treatment modalities such as TTP or Anti-vascular endothelial growth factor (Anti-VEGF) injections are also excluded.

Procedure and Steps

Patients received a Standard dose of 6 mg/m^2^ of body surface area (one study used double dose, one study used half dose) of verteporfin intravenously for 10 min (one study for 1 min, one study for 2 min). After 15 minutes (3 studies 5 min, one study 6 min) of infusion, exposure of 50 - 100 J/cm^2^ was applied at the radiance level of 600 mW/cm2 for 83 seconds (83s-186s) using laser and contact lens. Spot size was adjusted to include the entire lesion when possible, based on ophthalmoscopic appearance.

Data Extraction

Four co-authors independently extracted information from the included studies. The following information was extracted from each study: study design, number of study participants, average age of participants, gender of participants, PDT protocol used, number of PDT sessions applied, tumor location, baseline tumor characteristics including (Best Corrected Visual Acuity (BCVA), tumor thickness, tumor diameter, subretinal fluid (SRF), and exudative retinal detachment (ERD), follow-up duration, outcomes of tumor in last visit of follow-up, recurrence and reported complications (Table 1). Two different authors reviewed the extracted data and approved them.

**Table 1:** Studies included in the review Data has been represented as numbers, mean ± SD N/A: Not applicable; SD: Standard deviation

Author	Year	Country	Study design	NO. of patients	Average Age	Gender (N of Males)	Method of treatment	
							Dose protocol	Session Min-Max (Mean)
Jurklies B et al. [10]	2003	Germany	Prospective case-series	15	51	11	Standard Protocol	1-5
Vicuna-Kojchen J et al. [12]	2006	Isreal	Prospective case-series	9	55.7 ± 15.2	2	Standard Protocol	1 - 3
Madreperla SA et al. [14]	2001	N/A	Case series	3	58	2	Standard Protocol	1
Pérez-González D et al. [15]	2022	Isreal	Case-series	3	51	2	Half-Dosage Protocol	1
Elizalde J et al. [16]	2012	Spain	Retrospective case series	9	N/A	N/A	Standard Protocol	1-5
Zhang Y et al. [17]	2010	China	Retrospective case series	25	41 ± 7.8	17	Standard Protocol	1-2
Michels S et al. [18]	2005	Austria	Prospective case series	15	N/A	N/A	Standard Protocol	1-4
Robertson DM et al. [19]	2022	USA	Case series	3	58.33 ± 8.9	3	Standard Protocol	1-2
Lee JH et al. [20]	2017	Korea	Retrospective case series	17	All = 51.60 ± 6.55, Standard Dose (n = 7) = 50.57 ± 14.12, Double Dose (n = 10) = 51.60 ± 6.55	7 in standard-dose group, 7 in double-dose group	Standard Dose Protocol (7 patients), Double Dose (10 patients)	N/A
Verbraak FD et al. [21]	2003	Netherlands	Retrospective case series	11	47.9	8	Standard Protocol	1-2
Schmidt-Erfurth UM et al. [22]	2002	Germany	Prospective interventional case series	15	53	N/A	Standard Protocol	1-4
Blasi MA et al. [23]	2010	Italy	Prospective interventional case series	25	53.5	13	Standard Protocol	1 – 2
Porrini G et al. [24]	2003	Italy	Prospective case series	10	Range: 38 – 64	6	Standard Protocol	1-3
Gupta M et al. [26]	2004	UK	Case series	2	56	1	Standard Protocol	1 - 2
Ho YF et al. [27]	2018	Taiwan	Retrospective case series	9	46.5	8	Standard Protocol	1-2
Jamison A et al. [28]	2018	Scotland	Retrospective cohort	17	56	11	Standard Protocol	1-2
Wang M et al. [29]	2014	China	Retrospective study	27	44.54 ± 13.2	18	Standard Protocol	N/A
Verbraak FD et al. [30]	2006	Netherlands	Prospective non-randomized clinical study	6	46.5	4	Standard Protocol	1-3

Quality and Risk of Bias Assessment

The quality and risk of bias assessment of all 18 included studies [12,14-30] was conducted using the New Castle Ottawa scale for cross-sectional and cohort studies. Randomized controlled trials were evaluated using the Cochrane collaboration tool.

Results

Using pre-stated mesh terms, four databases were used to identify 262 registers. A total of 125 articles were removed before screening due to duplications, 141 studies were screened, 91 were excluded by humans, and no automation tools were used. The remaining 60 were assessed for full retrieval, where 10 were not recovered, the remaining 50 were evaluated for intervention and outcome, and 32 were excluded because they were irrelevant. Ultimately, the review encompassed 18 eligible articles (Figure 1).

All 18 eligible articles underwent a double independent review. Articles were published between the years 2001 [14] to 2022 [15], and most of the articles were published before 2010 [12, 16-30]. An overall 221 cases were included in the 18 articles. Most of the studies assessed CCH involving the central of the fovea, and CCH that does not involve the central of the fovea. The standard protocol was used in all included articles except for two studies, one of which used a half-dose protocol, and the other compared standard dose with double dose with an average number of sessions ranging from 1 to 2 sessions. At baseline assessment, the mean Pre-treatment BCVA ranged from 20/43 to 20/220, with five cases reporting counting finger vision. Different tumor thicknesses (mean range 0.6 to 4.1 mm) were reported, with a mean diameter ranging from 2.7 to 8.79 mm. CCH associated with SRF was reported among 14 cases, and ERD among nine cases where others lacked relative information. The duration of follow-up with patients ranged from 1 to 106 months, with high variability among the included articles. During the last visit, the mean BCVA ranged from 20/20 to 20/80, with only two cases reporting counting fingers. In the follow-up visit, tumor thickness showed atrophy with a mean range of 0 to 2.3mm; one failed treatment study showed 3.46 mm.

Furthermore, the diameter during follow-up ranged from total flattening of the tumor to 4.8mm; only two studies ranged from 6mm to 8mm amongst the studies that reported the effect on thickness and diameter. Cases showed resolution of SRF in 14 studies among the studies that reported the effect on SRFs. Additionally, 8 studies reported the Resolution of ERD; one study only showed no enhanced exudative reaction, among the studies that reported the effect on ERD (Table 2-3).

**Table 2:** Tumor location and characteristics before treatment Data has been represented as numbers N/A: Not applicable; CF: Counting fingers; BCVA: Best Corrected Visual Acuity; SRF: Subretinal Fluid; ERD: Exudative Retinal detachment; SD: Standard deviation

Author	Tumor location		Tumor characteristics before treatment and baseline BCVA				
	Number of patients with involvement in the central fovea	Number of patients without involvement in the central fovea	Mean BCVA	Mean tumor thickness	Mean tumor diameter	Presence of SRF	Presence of ERD
Jurklies B et al. [10]	0	9	20/50, except 2 patients with CF	2.7mm	8.6*8.0mm	N/A	N/A
Vicuna-Kojchen J et al. [12]	4	5	20/43	3.7mm	6.80mm	Present in 9 patients	Present in 9 patients
Madreperla SA et al. [14]	18	7	20/220	3.2mm	7.9mm	Present	Present in 25 patients
Pérez-González D et al. [15]	0	3	20/56	2.8mm	N/A	Present	N/A
Elizalde J et al. [16]	0	2	20/63	2.4mm	7.5mm	Present	N/A
Zhang Y et al. [17]	3	12	20/63	3.8mm	N/A	Present in 15 patients	Present in 5 patients
Michels S et al. [18]	1	2	20/100	2.84mm	7.5*4, 7*7.5, 9*8mm	Present in 1 patient	Present in 3 patients
Robertson DM et al. [19]	6	11	Standard dose group: 20/97. Double dose group: 20/66	Standard dose group: 2.62mm. Double dose group: 3.63mm	Standard dose group: 7.82mm. Double dose group: 8.79mm	Present	N/A
Lee JH et al. [20]	4	7	20/40	2.8mm	N/A	Present	Present
Verbraak FD et al. [21]	3	12	20/125	3.8mm	N/A	Present	present
Schmidt-Erfurth UM et al. [22]	0	25	20/57	3.35mm	N/A	N/A	N/A
Blasi MA et al. [23]	5	5	20/90	2.90mm	N/A	N/A	Present in 8 patients
Porrini G et al. [24]	12	5	20/63	0.6mm	N/A	Present	N/A
Gupta M et al. [26]	7	8	20/60	N/A	2.7mm	N/A	Present
Ho YF et al. [27]	2	4	20/25	N/A	3mm	Present	Present
Jamison A et al. [28]	0	27	Successfully treated: 20/50. Failed treatment: 20/140	Successfully treated 2.46mm. Failed treatment: 3.81mm	N/A	Present	N/A
Wang M et al. [29]	0	3	20/186	N/A	7.2mm	Present	N/A
Verbraak FD et al. [30]	0	9	20/100	4.1mm	N/A	N/A	N/A

**Table 3:** Tumor characteristics, recurrence & complications after treatment and follow-up Data has been represented as numbers, percentages N/A: Not applicable; CF: Counting fingers; BCVA: Best Corrected Visual Acuity; RD: Retinal detachment; SRF: Subretinal Fluid; ERD: Exudative Retinal detachment; RPE: Retinal pigment epithelium

Author	Tumor characteristics BCVA After treatment and follow-up					Follow up duration Min-Max	Recurrence	complications
	Mean BCVA	Mean Tumor Thickness	Mean tumor Diameter	Presence/ Resolution of Subretinal Fluid	Presence / Resolution of Exudative RD			
Jurklies B et al. [10]	20/32	N/A	1.3 mm	N/A	Resolution	2-24 months	N/A	NO
Vicuna-Kojchen J et al. [12]	20/50, except 2 patients with CF	0.8mm	N/A	8 Patients	N/A	6 - 24 months	N/A	Transient visual disturbances in 2 patients
Madreperla SA et al. [14]	20/24	N/A	N/A	Resolution	N/A	3-9 months	N/A	No
Pérez-González D et al. [15]	20/53	1 patient: 1.6 mm, 2 patients: significant shrinkage of tumor	Significant shrinkage	Resolution	N/A	12-28 months	N/A	NO
Elizalde J et al. [16]	20/28	2.3mm	N/A	8 Patients	N/A	7-67 months	SRF recurrence in 2 patients	Cystic degeneration of the retina (1 patient), RPE metaplasia (2 patients), RPE atrophy and microcystic degeneration (5 patients)
Zhang Y et al. [17]	20/63	1.3	4.8mm	Resolution	Resolution	12-60 months	N/A	No
Michels S et al. [18]	20/32	N/A	N/A	Resolution	N/A	12-66 months	N/A	Focal loss of choroid and retinal pigment epithelium
Robertson DM et al. [19]	20/80	2.1mm	N/A	N/A	Resolution	6 week - 16 month	NO	NO
Lee JH et al. [20]	20/69 (Standard Dose), 20/63 (Double Dose)	Standard-Dose group: 2.11mm. Double-Dose group: 3.01mm	Standard-Dose group: 6.05mm. Double-Dose group: 8mm	Standard-Dose group: Resolution in 4 patients. Double-Dose group: Resolution in 8 patients	N/A	12 months	Standard-Dose group: 2 patients. Double-Dose group: None	No
Verbraak FD et al. [21]	20/32	0mm	flattening of the tumor	Resolution	Resolution	3-22 months	NO	N/A
Schmidt-Erfurth UM et al. [22]	20/80	0.6mm	flattening of the tumor	Resolution	Resolution	3-24 MONTH	NO	Focal choroidal atrophy after the third treatment session
Blasi MA et al. [23]	20/20	0.80mm	N/A	Resolution	Resolved in 88% of eyes	73.5 ± 12.6 months	NO	NO
Porrini G et al. [24]	20/30	0.59mm	N/A	N/A	Resolution	7 to 16 months.	NO	Intraretinal edema in 2 cases
Gupta M et al. [26]	20/30	Total atrophy (0 mm)	7.5mm	Resolution	N/A	4-12 months	N/A	No
Ho YF et al. [27]	20/37	2.16mm	N/A	N/A	N/A	N/A	1 case	Tumor regrowth, Macular atrophy
Jamison A et al. [28]	20/25	0.3mm	N/A	Resolution	N/A	2-106 months	N/A	no
Wang M et al. [29]	Successful treatment: 20/40 failed treatment: 20/105	Successfully treated: 1.9mm, failed treatment: 3.46mm	N/A	Resolution	Resolved in 94% of eyes	Min 2 years	N/A	NO
Verbraak FD et al. [30]	20/20	N/A	0.4mm	Resolution	Resolution	18–29 months	NO	NO

Discussion

Choroidal hemangioma is a vascular tumor that is extremely uncommon and primarily affects the eye's choroid. It can be circumscribed or diffuse. The circumscribed type is typically a solitary tumor not associated with other ocular or systemic conditions. The diffuse type, on the other hand, is generally part of the Sturge-Weber syndrome. Choroidal hemangiomas are usually diagnosed between the second and fourth decade of life. ERD is an unfortunate consequence of CCH that has a severe impact on vision [2,31].

Treatment of choroidal hemangioma aims to minimize or eliminate visual impairment caused by SRF or foveal distortion due to the underlying tumor. As the haemangioma is generally located at the posterior pole, it is crucial to minimize injuries to the overlying portion of the retina to preserve visual acuity. A variety of techniques have been performed to treat the tumor, including argon laser photocoagulation [32], cryotherapy [33], external beam radiation therapy [34], proton beam radiation therapy [35], episcleral plaque radiation therapy [36] and TTT [37]. Although all of these treatment options have been found to stabilize or improve visual acuity to varying degrees, the significant drawback with each modality has been the risk of damage to the overlying retina.

Most of the reviewed articles are prospective case series studies conducted during a wide period interval (from 2001 to 2022). CCH involving the foveal center and CCH not involving the center of the fovea were evaluated in all included studies. All studies used standard protocol that managed tumors with different thicknesses except two studies (one used half dose, and the other compared standard dose with double dose). Furthermore, the study showed high variability regarding the follow-up intervals of the included cases. In the last follow-up, most studies showed improvement in the mean BCVA, Tumor thickness, and diameter [30]. Most studies reported that SRF and ERD showed resolution [23,24]. The majority of studies showed no tumor recurrence. Preliminary studies employing different treatment modalities have exhibited promising outcomes with PDT [38-42].

The former treatment options used for symptomatic CCH have proven efficacy. However, the recurrence rate is common, and if the fovea is affected, there is a possibility of a loss of vision [43-45]. Photocoagulation is one of the available modalities of management. However, this option only treats the surface of the hemangioma and cannot eliminate it. Recurrence of leakage is common, leading to progressive vision loss [44, 45]. PDT is a treatment that can cause targeted damage to vascular endothelial cells while leaving other retinal structures intact. While PDT has shown success in treating choroidal neovascularization, it is unclear whether it works similarly for central choroidal neovascularization, as no histopathologic studies have confirmed this [9,46].

According to Shields et al. [11], out of a 200 diagnosed cases group, 52% had a final visual acuity of 20/200 or worse. Patients with poor visual acuity at the time of presentation and those with symptoms of metamorphopsia, multi-quadrant SRF, and chronic RPE changes were at increased risk of developing poor visual acuity.

Shields CL et al. in 2020 [47] compared pre-PDT versus PDT Era in 458 cases and found that tumor basal diameter and thickness and fluorescein and indocyanine green angiography were similar in the 2 eras. On the other hand, patients in the PDT era demonstrated better mean logarithm of the minimum angle of resolution visual acuity of 1.28 vs 0.51 (Snellen equivalent 20/400 vs 20/63). The final visual acuity was ≥20/40 for those with entering vision of ≥20/40 (59.6% vs. 74.7%) and for those with entering vision of 20/50-20/200 (25.4% vs 47.3%).

Regarding adverse events and complications, only six studies showed complications, including RPE atrophy and microcystic degeneration (6 patients), Macular atrophy (3 patients), Transient visual disturbances (2 patients), RPE metaplasia (2 patients) and tumor regrowth (1 patient), two studies with large tumors after use of multiple PDT sessions showed focal loss of the choroid and RPE. Most studies did not show any serious adverse events regarding safety among the included cases. Most studies show no Recurrence (those whose report Recurrence), with only five patients in three studies having recurrence. It is believed that the standard AMD protocol, which employs infusion of a longer duration than modified protocols, may lower the risk of chorioretinal complications. However, Landau et al. [40] reported a case of temporary choroidal effusion and perifoveal hemorrhage, leading to vision loss in a patient treated with the standard AMD protocol.

## Conclusions

This systemic review discussed PDT's effectiveness and safety in managing CCH. It is effective in inducing tumor regression in diameter and thickness. We also demonstrated that the SRF and the ERD showed complete resolution. Additionally, we also reported significant improvement in BCVA. Most studies show no adverse events or serious side effects were reported, which coincides with the literature findings. However, some studies reported side effects, including atrophy, microcystic degeneration, Macular atrophy, Transient visual disturbances, RPE metaplasia, and tumor regrowth. Further studies focusing on the efficacy of PDT in comparison to other treatment modalities are needed.
